# Characterization of sarcoma topography in Li-Fraumeni syndrome

**DOI:** 10.3389/fonc.2024.1415636

**Published:** 2024-11-07

**Authors:** Karin J. Brockman, Mone’t B. Thompson, Lisa Mirabello, Sharon A. Savage, Ashkan Malayeri, Jessica N. Hatton, Payal P. Khincha

**Affiliations:** ^1^ Division of Cancer Epidemiology and Genetics, National Cancer Institute, National Institutes of Health, Bethesda, MD, United States; ^2^ Department of Pediatric Hematology/Oncology, Walter Reed National Military Medical Center, Bethesda, MD, United States; ^3^ Radiology and Imaging Sciences, National Institutes of Health, Bethesda, MD, United States

**Keywords:** Li-Fraumeni syndrome, TP53, sarcoma, whole body MRI, screening

## Abstract

**Introduction:**

Li-Fraumeni syndrome (LFS) is a hereditary cancer predisposition syndrome primarily caused by germline *TP53* pathogenic/likely pathogenic (P/LP) variants. Soft tissue and bone sarcomas are among the most frequently occurring of the many LFS-associated cancer types. Cancer screening recommendations for LFS are centered around annual whole-body MRI (wbMRI), the interpretation of which can be challenging. This study aims to characterize sarcoma topography in LFS.

**Methods:**

Study subjects included individuals from clinically and genetically ascertained cohorts of germline *TP53* variant-carriers, namely the National Cancer Institute’s LFS longitudinal cohort study (NCI-LFS), the NCI Genetic Epidemiology of Osteosarcoma (NCI-GEO) study, and the germline *TP53* Database.

**Results:**

Data was aggregated for a total of 160 sarcomas that had detailed topography available. Abdominal sarcomas and extremity osteosarcomas were among the most frequent locations of sarcomas. Chi-squared analyses showed no statistical differences in sarcoma topography based on age (pediatric vs adult) or sex (male vs female). A case series of sarcomas from the NCI-LFS study highlights the diagnostic challenges due to topography-related imaging.

**Discussion:**

While LFS-related sarcomas frequently occur in expected locations such as the extremities, they also occur in less typical sites, leading to difficulties in discerning between differential diagnoses on wbMRI and imaging. Prospective collection of detailed cancer topography in individuals with LFS will further aid in recommendations for radiologic interpretation and personalized screening in individuals with LFS.

## 1 Introduction

Li-Fraumeni syndrome (LFS) is a hereditary cancer predisposition syndrome primarily caused by germline pathogenic/likely pathogenic (P/LP) *TP53* variants. Risks for virtually all cancer types are extremely elevated in individuals with LFS, with overall 24-fold higher lifetime cancer incidence compared with the general population, and a high risk of multiple primary malignancies (1). The most frequently occurring ‘core’ cancers include pre-menopausal breast, soft tissue sarcoma (STS), brain, adrenocortical carcinoma, and osteosarcoma (1–3). Since the discovery of LFS in 1969, sarcomas have been a hallmark LFS-associated cancer, currently described as the most common first cancer in males with LFS and the second most common in females (1–3).

STS and osteosarcoma occur approximately 200 and 700 times more often, respectively, in individuals with LFS than in the general population (1). Despite significantly increased prevalence, the types of sarcoma diagnoses by age of onset mirror temporal patterns in the general population with rhabdomyosarcoma (RMS) most commonly diagnosed in young children, fibrosarcoma and osteosarcoma in children and young adults, malignant fibrous histiocytoma and liposarcoma diagnosed in childhood through middle adulthood, and leiomyosarcoma (LMS) diagnosed primarily in adults (4, 5). Gastrointestinal stromal tumors (GIST), Ewing sarcoma, and desmoid tumors do not occur at increased rates in LFS (5–9).

Given the high risk and heterogenous cancer types, individuals with LFS are subjected from an early age to multi-modal, high frequency cancer screening centered around annual whole-body MRI (wbMRI). In addition to wbMRI, children undergo annual brain MRI and quarterly abdominal ultrasounds; adults undergo annual brain MRI, dermatology screening, regular endoscopy and colonoscopy, as well as breast MRI and mammogram for females (10, 11). wbMRI has been established as a successful mode of early cancer detection and integral to cancer screening in LFS. However, it is associated with a high false positive rate and lack of standardization of technical protocol (12–16). Interpretation of wbMRI is difficult for many reasons, including being radiologist-dependent and the fact that early cancers may be very small and can occur anywhere in the body. False positive screens, which may lead to unnecessary intervention or anxiety, and false negative screens, which may result in a delayed or missed cancer diagnosis, have negative consequences (9, 14, 17, 18). The Oncologically Relevant Findings Reporting and Data System (ONCO-RADS) gives guidelines for interpreting wbMRI, including on patients receiving screening for cancer predisposition syndromes. ONCO-RADS recommends an interpretation template that is based on regional location of tumors that are identified in the screening (19). With the high risk for false positives/negatives and known importance of location in wbMRI interpretation, further understanding of sarcoma distribution in patients with LFS could be beneficial in wbMRI screening.

While a range of literature exists on sarcomas within LFS and on wbMRI surveillance, description of the bodily distribution of sarcomas within LFS is scarce. We aim to further characterize the frequency of topographical locations of LFS-associated sarcoma in cohorts of clinically or genetically ascertained LFS with germline *TP53* variants, highlighting both common and uncommon sites of sarcomas.

## 2 Methods

### 2.1 *TP53* variant designation

Germline *TP53* variants in all participants were classified as P/LP based on their designation in the ClinVar database and/or based on the recent ClinGen TP53 VCEP variant classification guidelines (20). Variants with inconclusive classification and variants of unknown significance were excluded.

### 2.2 Participant resources

Individuals in these resources meeting all three of the following criteria were included in the analysis: 1) confirmed P/LP germline variant in *TP53*, 2) primary sarcoma diagnosis, 3) description of location of sarcoma diagnosis. Sarcomas were then grouped under the topographical categories of head and neck, chest, breast, upper extremity, abdomen, pelvis, genitourinary, and lower extremity.

#### 2.2.1 NCI-longitudinal LFS study

Data for this retrospective cohort study was gathered from patients enrolled in the National Cancer Institute’s institutional review board-approved longitudinal Li-Fraumeni Syndrome study (hereafter called NCI-LFS) (NCT01443468). Participants or their legal guardians provided written, informed consent, completed questionnaires regarding personal and medical family history, and provided medical records for confirmation of genetic testing and cancer diagnoses (21). A subset of these individuals received annual cancer screening wbMRI at the NIH Clinical Center as part of the screening arm of the study (21).

#### 2.2.2 NCI-genetic epidemiology of osteosarcoma study

The National Cancer Institute’s Genetic Epidemiology of Osteosarcoma study (hereafter called NCI-GEO) includes osteosarcoma cases complete with data on osteosarcoma topography, unselected for family history, compiled from 10 participating international sites, primarily pediatric in nature (22). All participating subjects provided informed consent. Germline whole-exome sequencing (WES) of 1,004 osteosarcoma cases and targeted *TP53* sequencing of 765 osteosarcoma was previously performed at NCI and published (270 cases had both WES and targeting sequencing) (22). The cases that were identified to have a *TP53* P/LP variant were included here.

#### 2.2.3 Germline *TP53* database

The *TP53* Database is a publicly available database that has been an essential resource for understanding the phenotypic characteristics of *TP53* mutations including temporal patterns in previous studies (4, 23). The *TP53* germline variants phenotype file was obtained from the *TP53* Database (R20, July 2019): https://*TP53*.isb-cgc.org. Individuals with confirmed P/LP variants and at least one sarcoma diagnosis were included.

### 2.3 Statistical analysis

Chi-squared tests were used to compare differences in sarcoma topography stratified by age, and by sex. Analyses were performed using Microsoft Excel.

## 3 Results

### 3.1 Participant and sarcoma characteristics

#### 3.1.1 NCI-LFS cohort

The NCI-LFS cohort included 507 individuals with P/LP *TP53* variants. Of these, 171 sarcomas were diagnosed in 127 individuals. Specific topographical location was reported in 115 sarcomas, in 85 individuals (**Table 1** and **Supplementary Table 1**). Nearly 80% of sarcoma diagnoses were confirmed by medical record review (n=91, 79%), the other 21% were collected through participant self-report. Morphologies of these sarcomas in order of frequency included leiomyosarcoma (n=49, 42.6%), sarcoma, NOS (n=17, 14.8%), osteosarcoma (n=14, 12.2%), phyllodes tumor and spindle cell sarcoma (n=8, 7% each), rhabdomyosarcoma (n=6, 5.2%), liposarcoma (n=3, 2.6%), fibromyxosarcoma and myofibroblastic sarcoma (n=2, 1.7% each), with angiomyosarcoma, dermatofibrosarcoma, embryonal sarcoma, fibrosarcoma, lymphangiosarcoma, stromal sarcoma, and chondrosarcoma making up less than 1% each (n=1, <1%) (**Table 2**).

**Table 1:** Demographics of sarcomas cohorts, summarizing the total number of sarcomas analyzed in each cohort and broken down by sex.

	NCI-LFS Cohort N(%)	NCI-GEO Cohort N(%)	TP53 Database Cohort N(%)
Sex - All Cancer Types			
Male	187 (30)	20 (44)	540 (32)
Female	442 (70)	25 (56)	1042 (61)
Unknown	0	0	113 (7)
Total	629	45	1695
Sex - Sarcomas Only			
Male	63 (37)	20 (44)	199 (40)
Female	108 (63)	25 (56)	245 (49)
Unknown	0	0	53 (11)
Total	171	45	497
Sex - Sarcomas with Available Topography			
Male	33 (29)	20 (44)	––
Female	82 (71)	25 (56)	––
Total	115	45	––
Age - Sarcomas with Available Topography			
Median age at diagnosis	39.9 years	13 years	––

**Table 2:** Sarcoma morphology distribution in the NCI-LFS cohort and germline *TP53* database.

Sarcoma Morphology	NCI-LFS (n)	*TP53* Database (n)
Leiomyosarcoma	49	45
Sarcoma, NOS	17	103
Osteosarcoma	14	190
Phyllodes tumor	8	0
Spindle cell sarcoma	8	10
Rhabdomyosarcoma	6	99
Liposarcoma	3	25
Fibromyxosarcoma	2	0
Myofibroblastic Sarcoma	2	0
Angiomyosarcoma	1	0
Dermatofibrosarcoma	1	0
Embryonal sarcoma	1	0
Fibrosarcoma	1	11
Lymphangiosarcoma	1	0
Stromal sarcoma	1	3
Myxosarcoma	0	2
Chondrosarcoma	0	9
**TOTAL**	**115**	**497**

Bold text denotes total sarcoma counts in each database.

**Figure 1:**
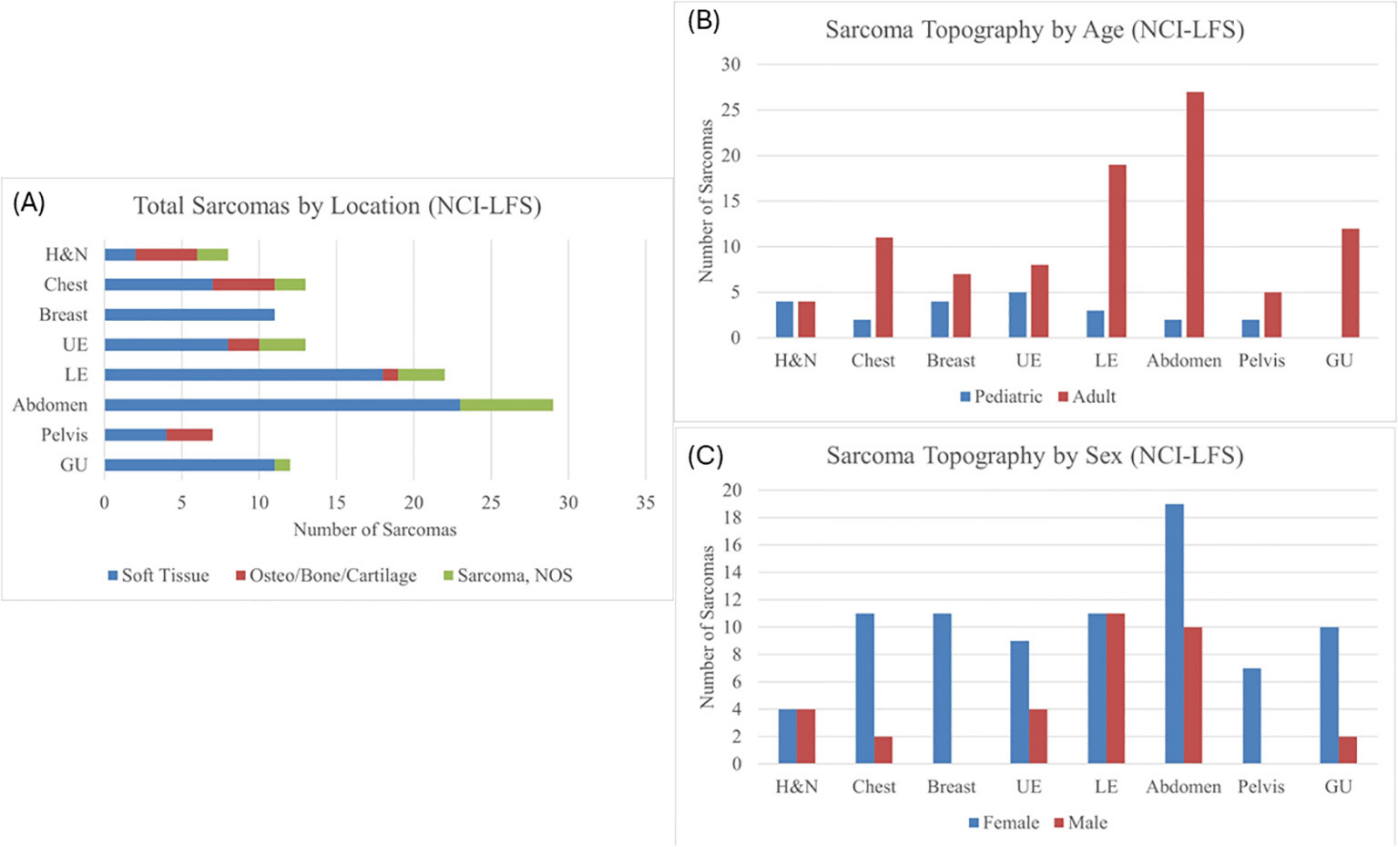
**(A)** Sarcoma topography distribution in the NCI-LFS Cohort, illustrating the proportion each location has of STS, osteosarcoma, and sarcoma, NOS. H&N, head and neck; UE, upper extremity; LE, lower extremity; GU, genitourinary; NOS, not otherwise specified. **(B)** Sarcoma topography in the NCI-LFS cohort stratified by age: pediatric (age <19 years) and adult (age ≥ 19 years). All chi-squared tests were not statistically significant with p>0.05. **(C)** Sarcoma topography in the NCI-LFS cohort stratified by sex. All chi-squared analyses resulted in a p-value >0.05.

Of the 115 sarcomas, 51 (44.3%) were the patient’s first cancer diagnosis, 29 (25.2%) were the second, and 35 (30.4%) were from patients who had previously had two or more cancers. STS formed the most common sarcoma diagnosis in 73% (n=84) sarcomas, the remainder being osteosarcoma in 12% (n=14) and sarcoma, NOS in 15% (n=17). The median age of diagnosis was 39.9 years (range 0.42-69) (**Table 1**). Majority (n=93, 81%) of the sarcomas were diagnosed during adulthood. Abdominal sarcomas were the most frequent STS (23/84, 27% of STS cases). Head and neck, and chest, were equally frequent for the osteosarcoma (4/14 and 29% each) (**Figure 1A**).

#### 3.1.2 NCI-GEO study

The NCI-GEO study included 1,499 osteosarcoma cases that underwent germline WES and/or *TP53* targeted sequencing, of which 45 osteosarcoma cases were determined to have 35 unique germline *TP53* variants that met inclusion criteria (**Supplementary Table 1**). Most cases (n=40, 89%) were diagnosed in the pediatric age. Median age at diagnosis was 13 years (range 7-40) (**Table 1**). Lower extremity was the most frequent location reported in 35 cases (77.8%).

#### 3.1.3 Germline *TP53* database

The *TP53* database contained 1,695 individuals with P/LP *TP53* variants. Of these, 497 sarcomas were diagnosed in 414 individuals (**Table 1**). Median age at sarcoma diagnosis was 16 years (range 0-69). Morphologies of these sarcomas in order of frequency included osteosarcoma (n=190, 38%), sarcoma, NOS (n=103, 20%), rhabdomyosarcoma (n=99, 20%), leiomyosarcoma (n=45, 9%), liposarcoma (n=25, 5%), fibrosarcoma (n=11, 2%), spindle cell sarcoma (n=10, 2%), chondrosarcoma (n=9, 2%), stromal sarcoma (n=3, 1%), and myxosarcoma (n=2, <1%) (**Table 2**). Unfortunately, the topography of these sarcomas was non-specific, resulting in them being excluded from statistical analyses. Predominantly reported topography included “Bones, joints, and articular cartilage of Other and unspecified sites”, “Connective, subcutaneous and other soft tissues”, “bones, joints, and articular cartilage of limbs”, and “other and unknown”.

### 3.2 Patterns of sarcoma topography by age and sex

Potential differences in sarcoma topography were evaluated by age and sex. Since the germline *TP53* database sarcomas did not have specific topography reported, they were excluded from this analysis.

Chi-squared analysis of the NCI-LFS data showed no statistically significant differences between pediatric and adult-aged sarcoma topography (**Figure 1B** and **Supplementary Table 2**). Furthermore, there was no statistically significant differences in sarcoma distribution between males and females (**Figure 1C** and **Supplementary Table 3**).

Combined analyses were performed for all sarcomas in both NCI-LFS and NCI-GEO data (n=160, **Supplementary Figure 1A**) and for all osteosarcomas (n=59) across the NCI-LFS and NCI-GEO cohorts. Chi-square analysis of the combined cohorts by age (pediatric *vs* adult at diagnosis) identified a statistically significant difference in lower extremity sarcomas (p=0.038, **Supplementary Figure 1B** and **Supplementary Table 4**); however, this significance did not exist when analysis was restricted to osteosarcomas alone (**Supplementary Table 5**). There were no statistically significant differences when comparing males and females (**Supplementary Figure 1C** and **Supplementary Table 6**).

## 4 Uncommon sarcoma topography in LFS

Though LFS-related sarcomas frequently occur in expected locations, they also occur in less typical sites, leading to difficulties in visualization and discerning between differential diagnoses. We present a case series of sarcomas from the NCI-LFS study, each of which highlight an uncommon topography and/or diagnostic wbMRI challenge.

### 4.1 Prostate sarcoma

**Figure 2:**
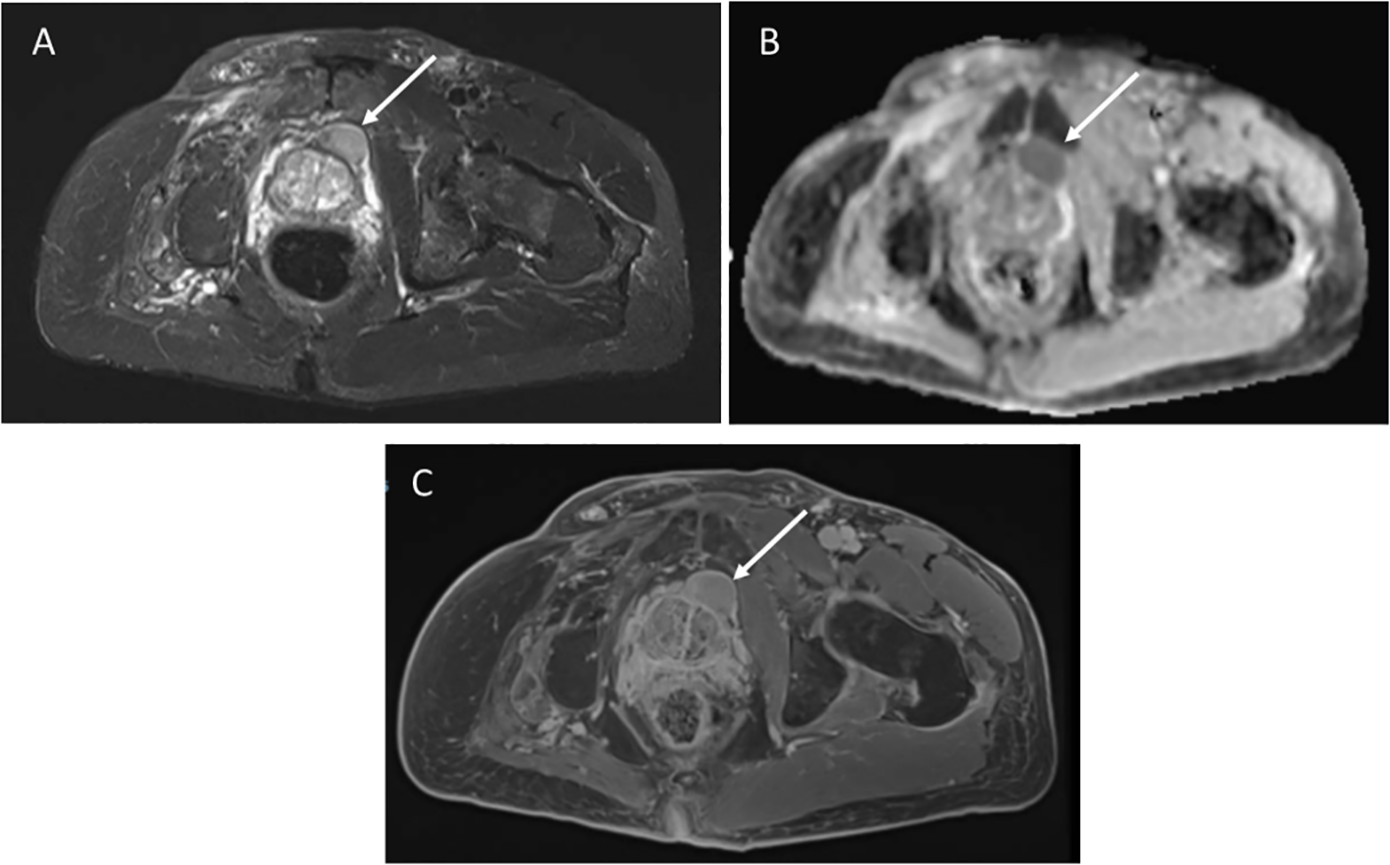
**(A)** Axial STIR image from whole body MRI study shows a mass anterior to the prostate gland, measuring 2.6 x1.5cm. **(B)** Apparent diffusion coefficient image shows restricted diffusion in the mass. **(C)** Axial post-contrast image demonstrates avid enhancement.

A 62-year-old male with germline *TP53* variant c.493C>T (p.Q165*) and a history of osteosarcoma of the left hip, esophageal cancer *in situ*, leiomyosarcoma of the right hand, leiomyosarcoma of the left upper arm with no prior radiation. He was found to have a mass anterior to his prostate of indeterminate nature on screening wbMRI. The mass continued to be stable in size over two years. An interval increase in the size of the mass was seen (2.6 x 1.5 cm) with restricted diffusion on diffusion-weighted imaging (DWI), and no changes seen in the prostate gland or seminal vesicles (**Figure 2**). Due to the concern for malignancy, PSA was measured and found to be normal at 3.69ng/mL (normal range 0-3.99ng/mL). Biopsy was recommended and confirmed the diagnosis of a high-grade prostate sarcoma. The sarcoma was successfully resected. He has since developed a non-Hodgkin’s lymphoma and a jejunal sarcoma.

### 4.2 Perirenal sarcoma

**Figure 3:**
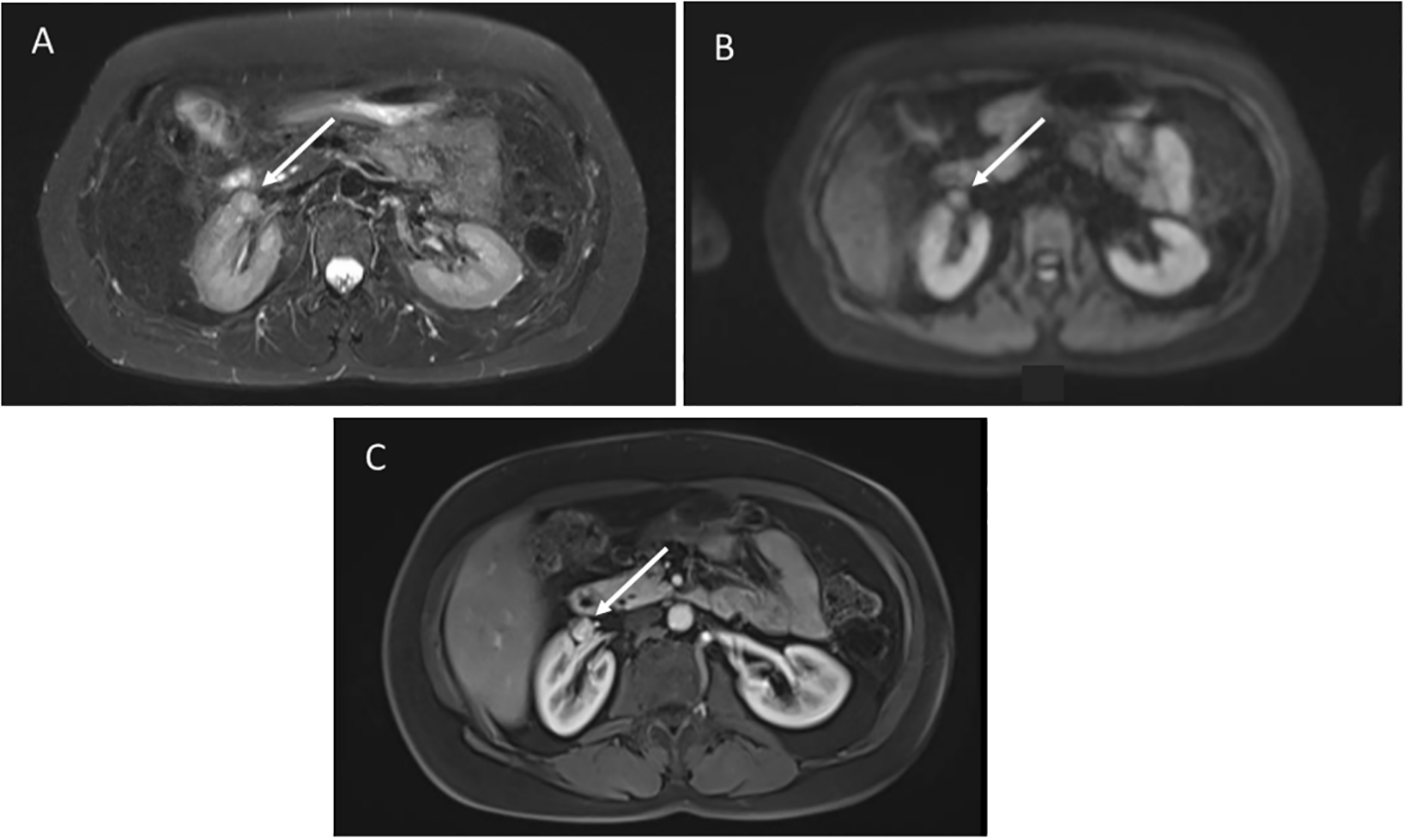
**(A)** Axial STIR images demonstrate a poorly defined mass anterior to the right kidney, not distinguishable from the hilar vessels. **(B)** Diffusion weighted imaging shows restricted diffusion. **(C)** Post-contrast images show hyperenhancement.

A 61-year-old female with germline *TP53* variant c.559 + 1G>A had a history of dermatofibrosarcoma of the right leg, invasive ductal carcinoma of the right breast, and alveolar adenocarcinoma of the left breast. Prior treatment included chemotherapy, and radiation for breast cancer. Screening wbMRI showed an enhancing 1.2 x 1.1 cm renal nodule identified along the anterior aspect of the right renal sinus (**Figure 3**). The lesion could not be further delineated from the renal hilar vessels was concerning for a potential venous varix or renal artery aneurysm. Ultrasound with doppler and arteriogram showed lack of blood flow creating the concern of an aneurysm thrombosed with either blood clot or tumor. Surgical resection confirmed pathology of well-differentiated leiomyosarcoma. On the same wbMRI she was found to have a right upper lobe lung nodule that was resected and diagnosed as a synchronous lung adenocarcinoma.

### 4.3 Retroperitoneal sarcoma

**Figure 4:**
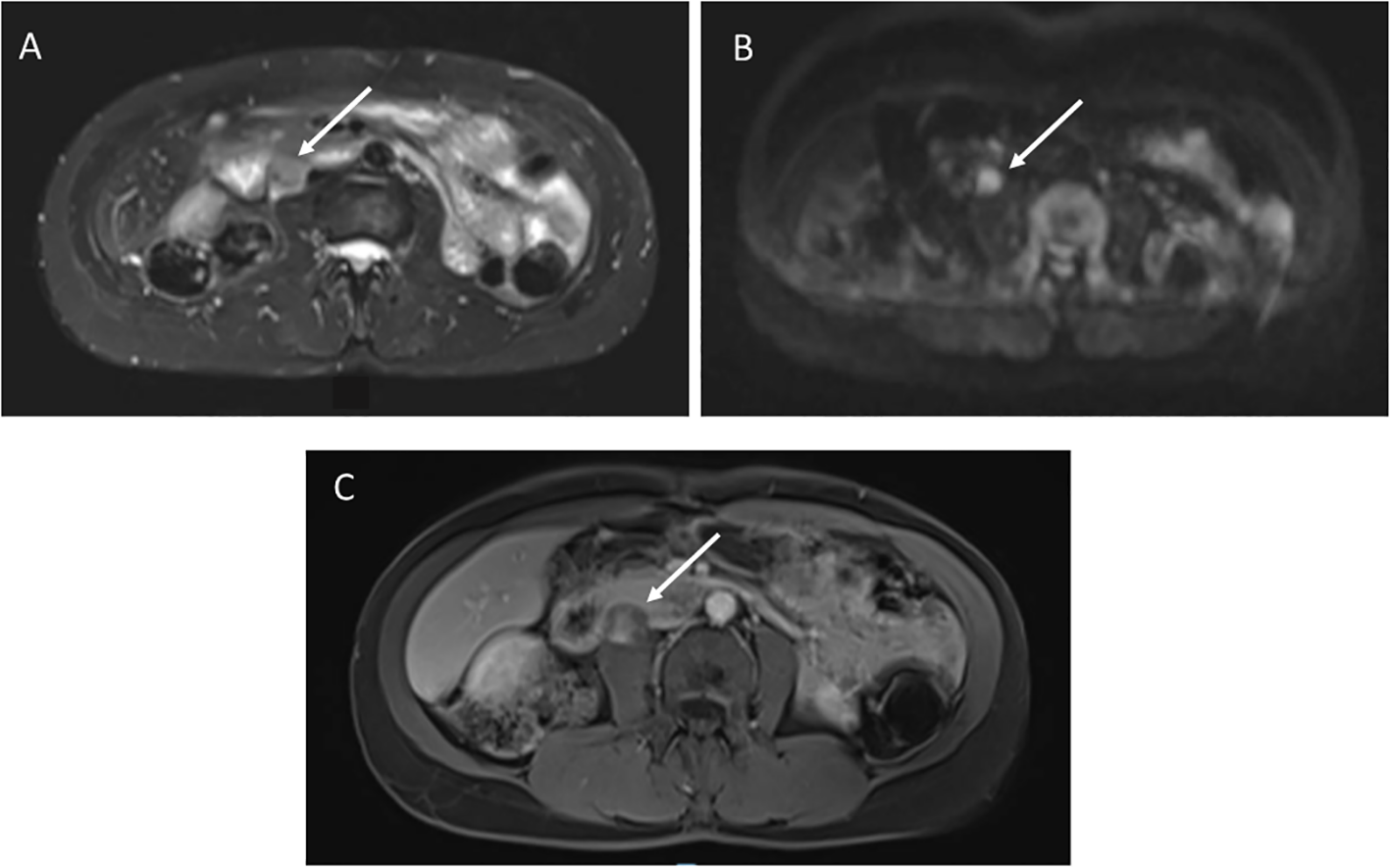
**(A)** STIR image demonstrates a 2 x 1.5 cm right retroperitoneal mass. **(B)** Diffusion weighted imaging shows restricted diffusion. **(C)** T1 post-contrast imaging showed heterogenous enhancement.

A 49-year-old female with germline *TP53* variant c.818G>A (p.R273H) had a history of infiltrating ductal carcinoma of the right breast and pulmonary adenocarcinoma in the right upper lobe, with no prior radiation therapy. Screening wbMRI showed a 2 x 1.5 cm hypoenhancing mass situated adjacent to the pancreas, duodenum, inferior vena cava and psoas muscle, difficult to visualize except appearing highly restricted on DWI imaging (**Figure 4**). An extended follow-up included a non-diagnostic computerized tomography (CT)-guided retroperitoneal biopsy. Open biopsy was subsequently performed that confirmed the pathology of a high grade leiomyosarcoma.

### 4.4 Chondroblastic osteosarcoma of left chest wall

**Figure 5:**
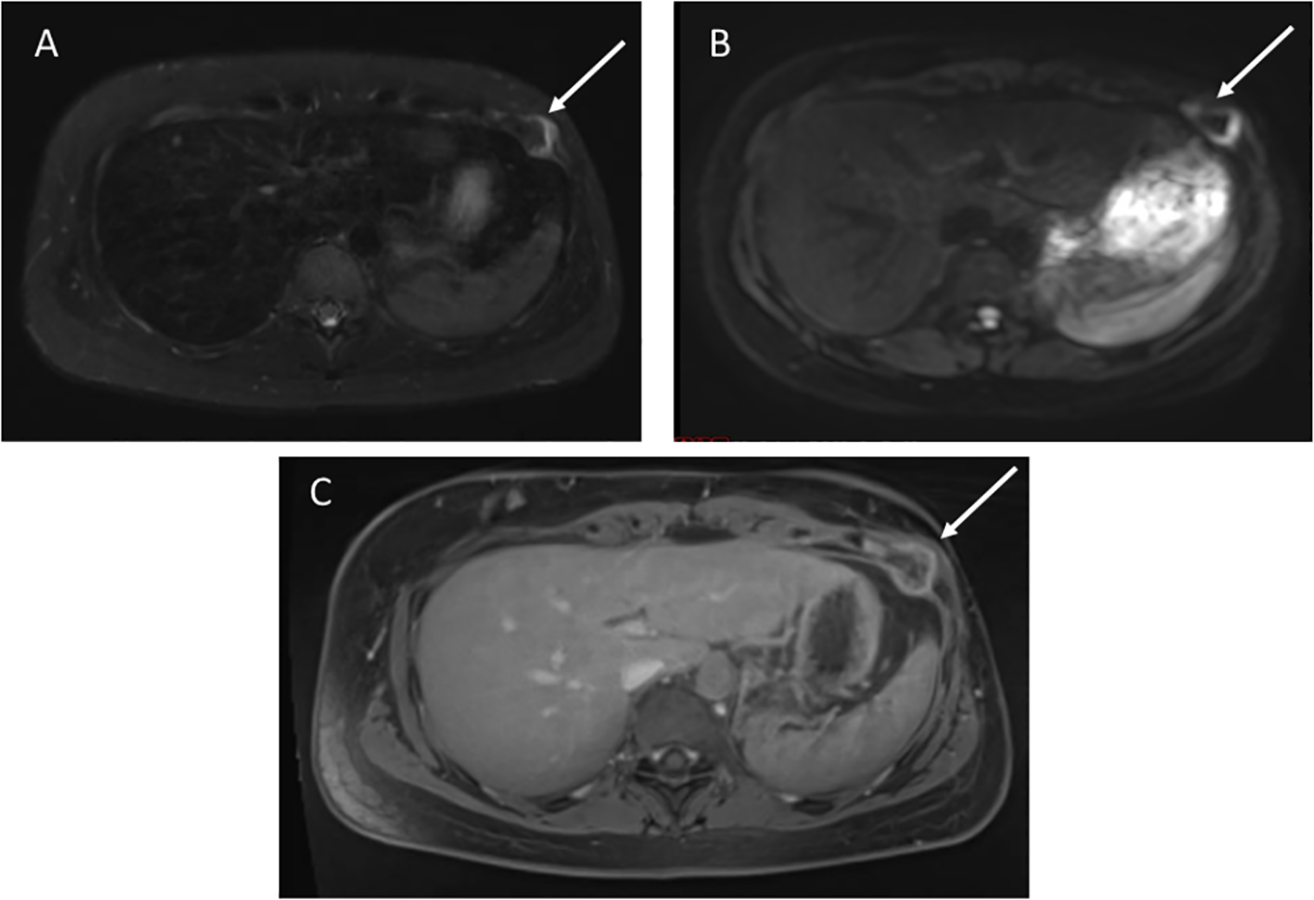
**(A)** Axial STIR image shows a 2.4 x 2.4 cm mass in the left anterior chest abdominal wall. **(B)** Diffusion weighted imaging demonstrates restricted diffusion. **(C)** T1 post-contrast imaging shows heterogenous enhancement.

A 28-year-old female with germline *TP53* variant c.189delinsAGA (p.P64fs) and a history of left breast sarcoma, treated with resection, chemotherapy and radiation. Screening wbMRI identified a 2.4 x 2.4 cm new indeterminate enhancing lesion along the left anterior abdominal wall that was initially concerning for trauma, but on further review of history was recommended to be biopsied (**Figure 5**). Ultrasound-guided biopsy confirmed the pathology of chondroblastic osteosarcoma of the left chest wall, treated with chemotherapy and surgical resection.

### 4.5 Gastric leiomyosarcoma

A 46-year-old female with germline *TP53* variant c.389T>C (p.L130P) had a history of multiple primary breast cancers, papillary thyroid carcinoma, and clear cell pancreatic endocrine neoplasm. She had no prior radiation therapy. Per screening recommendations, she underwent an upper endoscopy on which a small lesion was excised and confirmed to be a gastric leiomyosarcoma. wbMRI three months prior did not identify any abnormalities at the time. No further treatment other than repeat endoscopy surveillance was recommended, due to clear tumor margins.

## 5 Discussion

Sarcoma risk and incidence have been established to be much higher in LFS compared with the general population (1, 5). To date, published literature on LFS-associated sarcoma characterization, genotype-phenotype correlations and comparative stratification by age and sex have typically focused on sarcoma morphology (4, 5). There continues to be a scarcity of studies characterizing tumor topography in LFS, and other tumor predisposition syndromes. This study provides the first-known in-depth characterization of LFS-associated sarcoma topography, highlighting both common and uncommon sites of this core LFS cancer diagnosis.

Current cancer screening recommendations for LFS are universal regardless of prior cancer history, or germline *TP53* variant (10, 11). While wbMRI has been well established as an optimal screening modality in LFS, there are limitations including false positivity and lack of standardized protocols for wbMRI reporting (12, 24). Identification of sarcomas may be difficult due to the wide range of body sites and sizes they can occur in, along with the large number of imaging sequences to be reviewed by radiologists with each wbMRI. Maintaining a high suspicion of sarcoma with relevant follow up recommendations must be weighed against the risk of unnecessary intervention while utilizing such a comprehensive screening technology. Understanding the most frequent and most likely topography for sarcomas to occur may aid in understanding their risk profile and wbMRI interpretation, to ensure adequate clinical follow up.

Our study did not identify any statistical differences in sarcoma topographical distribution by age (pediatric vs. adult) or sex (male vs. female). Though a statistically significant difference was detected for the lower extremity sarcomas based on age in the combined NCI-LFS +NCI-GEO sarcoma cohort, this difference was absent from the NCI-LFS cohort analysis and the combined osteosarcoma-only analysis, suggesting artificial inflation of pediatric lower extremity cases due to the propensity of osteosarcomas to present in the distal femur and proximal tibia.

One significant limitation of the study was due to lack of identification of sarcoma location in diagnostic data. This was strikingly evident in the germline *TP53* database where specific topography was not available for close to 500 sarcomas. ICD-10 codes in clinical data are also not always specific to an anatomic location, but more to the involved tissue, as experienced in the NCI cohort data.

However, our data and case series do reinforce certain best practices for sarcoma screening in LFS. For instance, the retroperitoneal sarcoma described above was only visualized through DWI sequences, and gastrointestinal sarcomas such as the gastric leiomyosarcoma above may only be identified through more direct visualization, specifically colonoscopy or endoscopy, both of which are recommended in adults over 25 years of age with LFS (11). Additionally, the most common sarcoma location overall was the lower extremity, highlighting the importance for inclusivity of full-extremity visualization on LFS wbMRI protocols.

In conclusion, while current LFS screening protocols are comprehensive and promote early detection of malignancies, identification of sarcomas can prove challenging, due to limitations in imaging modality or interpretation. wbMRI protocols should be comprehensive, ideally including DWI imaging and completely visualizing extremities. As we head into the era of precision medicine, efforts are needed to evaluate possible tailored approaches to cancer screening in LFS. Our data show that distributive characterization is feasible with prospective comprehensive topographical data collection, allowing for possible consequent development of tailored screening approaches for sarcomas.

## Data Availability

Datasets related to this article will be shared directly with researchers whose research requests are consistent with study consent forms, after an appropriate data-sharing agreement has been established. Requests to access the datasets should be directed to the corresponding author.
