# Supplementary material for: Characterization of sarcoma topography in Li-Fraumeni syndrome
Source: Front Oncol. 2024 Nov 7;14:1415636. doi: 10.3389/fonc.2024.1415636 (PMC11578819; doi:10.3389/fonc.2024.1415636)
Supplement: Supplementary file 1 [file DataSheet1.docx]

Supplementary Tables

**Supplementary Table 1.** Germline *TP53* variants identified in individuals from the NCI-LFS and NCI-GEO cohorts included in the study.

| **Germline *TP53* Variant (c.)** | **Germline *TP53* Variant (p.)** | **Number of individuals with variant** | |
| --- | --- | --- | --- |
|  |  | **NCI-LFS** | **NCI-GEO** |
| c.?(del 5'UTR_3'UTR) | 5'UTR_3'UTR | 2 | 0 |
| c.?(del 5'UTR_exon 1) | p.? | 5 | 0 |
| c.451C>G | p.P151A | 0 | 1 |
| c.?(del exon1-8) | p.? | 4 | 0 |
| c.?(del prom_ex1) | p.? | 1 | 0 |
| c.1006G>T | p.E336* | 0 | 1 |
| c.1010G>A | p.R337H | 2 | 0 |
| c.1024C>T | p.R342* | 3 | 0 |
| c.1025G>C | p.R342P | 1 | 0 |
| c.1040C>A | p.A347D | 5 | 0 |
| c.159G>A | p.W53* | 2 | 0 |
| c.189delinsAGA | p.P64fs | 6 | 0 |
| c.227_279del | p.A76fs | 3 | 0 |
| c.-29+1G>T | p.? | 3 | 0 |
| c.331_365dup | p.T123fs | 1 | 0 |
| c.332T>C | p.L111P | 2 | 0 |
| c.365_366del | p.V122fs | 1 | 0 |
| c.375G>A | p.T125T | 2 | 0 |
| c.375G>C | p.T125T | 0 | 1 |
| c.389T>C | p.L130P | 1 | 0 |
| c.454_466del | p.P152fs | 0 | 1 |
| c.455C>T | p.P152L | 1 | 0 |
| c.473G>A | p.R158H | 5 | 0 |
| c.493C>T | p.Q165* | 6 | 0 |
| c.524G>A | p.R175H | 0 | 3 |
| c.530C>G | p.P177R | 0 | 1 |
| c.535C>T | p.H179Y | 1 | 0 |
| c.559+1G>A | p.? | 2 | 0 |
| c.569C>T | p.P190L | 1 | 0 |
| c.586C>T | p.R196* | 4 | 1 |
| c.636del | p.R213fs | 1 | 0 |
| c.637C>T | p.R213* | 2 | 0 |
| c.655C>T | p.P219S | 1 | 0 |
| c.659A>G | p.Y220C | 0 | 1 |
| c.660T>A | p.Y220* | 1 | 0 |
| c.711G>A | p.M237I | 3 | 0 |
| c.712T>A | p.C238S | 1 | 0 |
| c.713G>A | p.C238Y | 0 | 2 |
| c.724T>C | p.C242R | 0 | 1 |
| c.725G>A | p.C242Y | 0 | 1 |
| c.733G>A | p.G245S | 6 | 1 |
| c.736A>G | p.M246V | 7 | 0 |
| c.742C>T | p.R248W | 3 | 1 |
| c.743G>A | p.R248Q | 5 | 2 |
| c.817C>A | p.R273S | 0 | 1 |
| c.817C>T | p.R273C | 1 | 1 |
| c.818G>A | p.R273H | 6 | 5 |
| c.821T>C | p.V274A | 4 | 0 |
| c.832C>G | p.P278A | 0 | 1 |
| c.844C>T | p.R282W | 1 | 2 |
| c.856G>A | p.E286K | 0 | 1 |
| c.892G>T | p.E298* | 1 | 0 |
| c.916C>T | p.R306* | 2 | 1 |
| c.919+1G>A | p.? | 0 | 1 |
| c.920-1G>A | p.? | 1 | 0 |
| c.95_96+6delinsCTT | p.? | 2 | 0 |
| c.951del | p.Q317fs | 0 | 1 |
| c.?(del exon 1-11) | p.? | 3 | 0 |
| c.-29+1G>C | p.? | 0 | 1 |
| c.340_346dup | p.S116fs | 0 | 1 |
| c.358A>G | p.K120E | 0 | 2 |
| c.390_426del | p.N131fs | 0 | 1 |
| c.439del | p.V147fs | 0 | 1 |
| c.782+1G>A | p.? | 0 | 1 |
| c.782+2T>G | p.? | 0 | 1 |
| c.836G>A | p.G279E | 0 | 1 |
| c.880del | p.E294fs | 0 | 1 |
| c.902del | p.P301fs | 0 | 1 |
| c.902dup | p.G302fs | 0 | 1 |
| c.995T>G | p.I332S | 0 | 1 |

**Supplementary Table 2.** Statistical analyses of sarcoma topography in LFS compared by age in the NCI-LFS cohort (Chi-squared analysis, degrees of freedom (DoF) is 7).

| **NCI-LFS Cohort Only** | | | | | | |
| --- | --- | --- | --- | --- | --- | --- |
|  | Cohort Numbers | | Expected Numbers | |  |  |
| Location | Pediatric | Adult | Pediatric | Adult | x^2 | P-value (DoF = 7) |
| H&N | 4 | 4 | 1.5 | 6.5 | 4.93 | 0.67 |
| Chest | 2 | 11 | 2.5 | 10.5 | 0.12 | >0.99 |
| Breast | 4 | 7 | 2.1 | 8.9 | 2.11 | 0.95 |
| UE | 5 | 8 | 2.5 | 10.5 | 3.14 | 0.87 |
| LE | 3 | 19 | 4.2 | 17.8 | 0.43 | >0.99 |
| Abdomen | 2 | 27 | 5.6 | 23.5 | 2.81 | 0.9 |
| Pelvis | 2 | 5 | 1.3 | 5.7 | 0.40 | >0.99 |
| GU | 0 | 12 | 2.3 | 9.7 | 2.84 | 0.9 |

**Supplementary Table 3.** Statistical analyses of sarcoma topography in LFS compared by sex in the NCI-LFS cohort (Chi-squared analysis, degrees of freedom (DoF) is 7).

| **NCI-LFS Cohort Only** | | | | | | |
| --- | --- | --- | --- | --- | --- | --- |
|  | Cohort Numbers | | Expected Numbers | |  |  |
| Location | Female | Male | Female | Male | x^2 | P-value (DoF = 7) |
| H&N | 4 | 4 | 5.7 | 2.3 | 1.77 | 0.97 |
| Chest | 11 | 2 | 9.3 | 3.7 | 1.13 | 0.99 |
| Breast | 11 | 0 | 7.8 | 3.2 | 4.43 | 0.73 |
| UE | 9 | 4 | 9.3 | 3.7 | 0.03 | 1 |
| LE | 11 | 11 | 15.7 | 6.3 | 4.88 | 0.67 |
| Abdomen | 19 | 10 | 20.7 | 8.3 | 0.47 | >0.99 |
| Pelvis | 7 | 0 | 5.0 | 2.0 | 2.82 | 0.9 |
| GU | 10 | 2 | 8.6 | 3.4 | 0.85 | >0.99 |

**Supplementary Table 4.** Statistical analyses of sarcoma topography in LFS compared by sex in the combined NCI-LFS+NCI-GEO cohorts (Chi-squared analysis, degrees of freedom (DoF) is 7).

| **NCI-LFS+NCI-GEO Cohort** | | | | | | |
| --- | --- | --- | --- | --- | --- | --- |
|  | Cohort Numbers | | Expected Numbers | |  |  |
| Location | Pediatric | Adult | Adult | Pediatric | x^2 | P-value (DoF = 7) |
| H&N | 5 | 4 | 5.5 | 3.5 | 1.07 | 0.99 |
| Chest | 3 | 12 | 9.2 | 5.8 | 2.22 | 0.95 |
| Breast | 4 | 7 | 6.7 | 4.3 | 0.03 | 1.00 |
| UE | 7 | 9 | 9.8 | 6.2 | 0.17 | 0.99 |
| LE | 36 | 21 | 34.9 | 22.1 | 14.31 | **0.038** |
| Abdomen | 2 | 27 | 17.8 | 11.2 | 12.40 | 0.10 |
| Pelvis | 5 | 6 | 6.7 | 4.3 | 0.21 | 0.99 |
| GU | 0 | 12 | 7.4 | 4.7 | 7.59 | 0.38 |

**Supplementary Table 5.** Statistical analysis of osteosarcoma topography in the NCI-LFS + NCI-GEO cohorts (Chi-squared analysis, degrees of freedom (DoF) is 4).

| **Osteosarcoma Data only** | | | | | | |
| --- | --- | --- | --- | --- | --- | --- |
|  | Cohort Numbers | | Expected Numbers | |  |  |
| Location | Pediatric | Adult | Pediatric | Adult | x^2 | P-value (DoF = 4) |
| H&N | 2 | 3 | 3.8 | 1.2 | 3.63 | 0.46 |
| Chest | 2 | 4 | 4.6 | 1.4 | 6.11 | 0.19 |
| Breast | 0 | 0 | 0.0 | 0.0 | n/a |  |
| UE | 3 | 2 | 3.8 | 1.2 | 0.73 | 0.95 |
| LE | 33 | 3 | 27.5 | 8.5 | 4.71 | 0.32 |
| Abdomen | 0 | 0 | 0.0 | 0.0 | n/a |  |
| Pelvis | 5 | 2 | 5.3 | 1.7 | 0.09 | 0.99 |
| GU | 0 | 0 | 0.0 | 0.0 | n/a |  |

**Supplementary Table 6.** Statistical analysis of sarcoma topography by sex in the combined NCI-LFS + NCI-GEO cohorts. (Chi-squared analysis, degrees of freedom is 7).

| **NCI-LFS+NCI-GEO Cohort** | | | | | | |
| --- | --- | --- | --- | --- | --- | --- |
|  | Cohort Numbers | | Expected Numbers | |  |  |
| Location | Female | Male | Female | Male | x^2 | P-value (DoF = 7) |
| H&N | 4 | 5 | 6.0 | 3.0 | 2.04 | 0.96 |
| Chest | 12 | 3 | 10.0 | 5.0 | 1.17 | 0.99 |
| Breast | 11 | 0 | 7.4 | 3.6 | 5.45 | 0.6 |
| UE | 11 | 5 | 10.7 | 5.3 | 0.03 | 0.99 |
| LE | 30 | 27 | 38.1 | 18.9 | 5.22 | 0.65 |
| Abdomen | 19 | 10 | 19.4 | 9.6 | 0.02 | 0.99 |
| Pelvis | 10 | 1 | 7.4 | 3.6 | 2.87 | 0.89 |
| GU | 10 | 2 | 8.0 | 4.0 | 1.47 | 0.98 |

**Supplementary Figures**

**Supplementary Figure 1. (A)** Sarcoma topography distribution in the NCI-LFS and NCI-GEO cohorts, illustrating the proportion each location has of STS, osteosarcoma, and sarcoma, NOS. H&N = head and neck, UE = upper extremity, LE = lower extremity, GU = genitourinary, NOS= not otherwise specified. **(B)** Sarcoma topography in the combined NCI-LFS and NCI-GEO cohorts stratified by age: pediatric (age <19 years) and adult (age ≥ 19 years). *= statistically significant difference in lower extremity sarcomas (p=0.038). **(C)** Sarcoma topography in the combined NCI-LFS + NCI-GEO cohorts stratified by sex. All chi-squared analyses were not statistically significant with p-value >0.05.

**(A)**

**(B)**

**(C)**
